# An Intraoperative Ultrasound Evaluation of Axillary Lymph Nodes: Cassandra Predictive Models in Patients with Breast Cancer—A Multicentric Study

**DOI:** 10.3390/medicina60111806

**Published:** 2024-11-04

**Authors:** Simona Parisi, Francesco Saverio Lucido, Federico Maria Mongardini, Roberto Ruggiero, Francesca Fisone, Salvatore Tolone, Antonio Santoriello, Francesco Iovino, Domenico Parmeggiani, David Vagni, Loredana Cerbara, Ludovico Docimo, Claudio Gambardella

**Affiliations:** 1 Department of Advanced Medical and Surgical Sciences, Division of General, Oncological, Mini-Invasive and Obesity Surgery—University of Study of Campania “Luigi Vanvitelli”, 80136 Naples, Italy; francescosaverio.lucido@unicampania.it (F.S.L.); f.mongardini@gmail.com (F.M.M.); roberto.ruggiero@unicampania.it (R.R.); fisonefrancesca@gmail.com (F.F.); salvatore.tolone@unicampania.it (S.T.); domenico.parmeggiani@unicampania.it (D.P.); ludovico.docimo@unicampania.it (L.D.); claudio.gambardella2@unicampania.it (C.G.); 2 Breast Unit, Division of Surgery, Cobelli’s Hospital, Vallo della Lucania, 84078 Salerno, Italy; antonio.santoriello2@gmail.com; 3 Department of Traslational Sciences, Division of General, Oncological, Mini-Invasive and Obesity Surgery—University of Study of Campania “Luigi Vanvitelli”, 80136 Naples, Italy; francesco.iovino@unicampania.it; 4 National Research Council, Institute for Research and Biomedical Innovation, 98164 Messina, Italy; david.vagni@irib.cnr.it; 5 National Research Council, Institute for Research on Population and Social Policies (CNR-IRPPS), 00185 Rome, Italy; loredana.cerbara@cnr.it

**Keywords:** axillary staging, breast cancer, axillary ultrasound, axillary surgery, ultrasound score

## Abstract

*Background and Objectives:* Axillary lymph node (ALN) staging is crucial for the management of invasive breast cancer (BC). Although various radiological investigations are available, ultrasound (US) is the preferred tool for evaluating ALNs. Despite its immediacy, widespread use, and good predictive value, US is limited by intra- and inter-operator variability. This study aims to evaluate US and Elastosonography Shear Wave (SW-ES) parameters for ALN staging to develop a predictive model, named the Cassandra score (CS), to improve the interpretation of findings and standardize staging. *Materials and Methods:* Sixty-three women diagnosed with BC and treated at two Italian hospitals were enrolled in the study. A total of 529 lymph nodes were surgically removed, underwent intraoperative US examination, and were individually sent for a final histological analysis. The study aimed to establish a direct correlation between eight US-SWES features (margins, vascularity, roundness index (RI), loss of hilum fat, cortical thickness, shear-wave elastography hardness (SWEH), peripheral infiltration (PI), and hypoechoic appearance) and the histological outcome (benign vs. malignant). *Results:* Several statistical models were compared. PI was strongly correlated with malignant ALNs. An ROC analysis for Model A revealed an impressive AUC of 0.978 (S.E. = 0.007, *p* < 0.001), while in Model B, the cut-offs of SWEH and RI were modified to minimize the risk of false negatives (AUC of 0.973, S.E. = 0.009, *p* < 0.001). Model C used the same cut-offs as Model B, but excluded SWEH from the formula, to make the Cassandra model usable even if the US machine does not have SW-ES capability (AUC of 0.940, S.E. = 0.015, *p* < 0.001). A two-tiered model was finally set up, leveraging the strong predictive capabilities of SWEH and RI. In the first tier, only SWES and RI were evaluated: a positive result was predicted if both hardness and roundness were present (SWES > 137 kPa and RI < 1.55), and conversely, a negative result was predicted if both were absent (SWES < 137 kPa and RI > 1.55). In the second tier, if there was a mix of the results (SWES > 137 kPa and RI > 1.55 or SWES < 137 kPa and RI < 1.55), the algorithm in Model B was applied. The model demonstrated an overall prediction accuracy of 90.2% in the training set, 87.5% in the validation set, and 88.9% across the entire dataset. The NPV was notably high at 99.2% in the validation set. This model was named the Cassandra score (CS) and is proposed for the clinical management of BC patients. *Conclusion:* CS is a simple, non-invasive, fast, and reliable method that showed a PPV of 99.1% in the malignancy prediction of ALNs, potentially being also well suited for young sonographers.

## 1. Introduction

Axillary lymph node (ALN) staging plays a crucial role in the management of invasive breast cancer (BC), influencing the choice between surgery and neoadjuvant treatments. Besides imaging, disease evaluation currently involves the pathological examination of the primary tumor and cytology or histology of the axillary nodes when involvement is suspected [1].

Conversely, axillary lymph node dissection (ALND) was the predominantly recommended procedure for BC staging, but it was associated with significant comorbidities such as arm lymphedema, also demonstrating inconsistent benefits for BC survival. Sentinel lymph node biopsy (SLNB) has replaced ALND for axillary staging, proving to be less invasive but equally accurate. Numerous randomized studies have confirmed the feasibility and safety of SLNB, demonstrating encouraging results in reducing surgical complications. Currently, SLNB is strongly recommended by major guidelines when ALNs are not suspicious [2,3,4,5,6,7,8,9].

In this context, clinical and radiological assessment of ALN at the time of the diagnosis is crucial. Radiological false positives may preclude SLNB, leading to overtreatment and potentially surgical complications. Conversely, false negatives may result in unnecessary SLNB, exposing patients to radioactive trackers. Clinical examination, with a sensitivity value of up to 30%, is not considered a reliable tool. Radiological investigations, including mammography, which is the gold standard for BC screening, have limitations in evaluating the axilla. Magnetic Resonance Imaging (MRI), with its higher resolution power, is a second-level tool. However, it has drawbacks such as pulsation artifacts and incomplete axillary visualization. Computed Tomography (CT) is not commonly performed, indicated only in suspected advanced BC, with moderate sensitivity and low specificity for axillary staging [10,11,12,13]. Ultrasound (US) is the preferred imaging modality for evaluating axillary lymph nodes due to its low cost and easy availability. It more effectively defines lymph node morphology and identifies cortical morphologic changes better than MRI. US has a specificity of 88–98%, while sensitivity varies between 26 and 76%. Influential US features for ALN staging have been established. The guidelines of the European Federation of Societies for Ultrasound in Medicine and Biology (EFSUMB) suggest that elastosonography (ES) of superficial lymph nodes is a promising tool for axillary staging, verifying the increase in stiffness in malignant lymph nodes. Furthermore, the shear wave (SW) ES also has the characteristic of providing an objective measure of the stiffness of the lesions with evaluation in Pascal [14,15,16].

US and ES features have been investigated to improve axillary preoperative staging, but the evaluation of lymph node suspicion remains operator-dependent, affecting reliability. Therefore, a new scoring system can enhance objective predictability, reducing individual judgment and guiding less experienced operators.

The study aimed to design and validate an innovative differentiation model of benign and malignant ALNs, which we will call the Cassandra score (CS), by analyzing eight ultrasound–SW elastosonography (US-ES) features. The ultimate purpose of the study was to develop a simple algorithm for clinical decision making to improve the sensitivity and specificity of the diagnostic model and reduce the rate of unnecessary SLNB or more invasive treatments [17,18,19,20].

## 2. Materials and Methods

### 2.1. Study Design

This study is reported according to the STROBE statement for cohort studies [21]. A retrospective multicentric study was conducted to analyze the correlation between the innovative US-ES predictive models, the CS, and the presence of metastatic ALNs in BC patients. It was conducted according to the ethical principles stated in the Declaration of Helsinki. Written informed consent was obtained from all patients.

### 2.2. Study Setting and Study Population

Between January 2022 and January 2023, all the patients referred to the Surgical and Oncological Unit of Campania University “Vanvitelli” (Naples, Italy) and the Breast Surgery Unit of “Cobellis” Hospital (Salerno, Italy) for breast cancer (BC) were included in the study. Candidates were identified based on multidisciplinary indications for axillary lymph node dissection (ALND), specifically having at least one axillary lymph node suspected at preoperative ultrasound (US) and confirmed through core biopsy. The participants had refused or had contraindications to neoadjuvant therapy.

The inclusion criteria were as follows:

(1) BC confirmed through core biopsy.
(2) US examination conducted within 30 days before surgery.
(3) Indication for ALND.
(4) Single BC measuring less than 5 cm (cm).

Exclusion criteria were defined as

(1) Prior neoadjuvant treatment.
(2) Presence of multiple BCs.

A preoperative US-ES had been performed, and suspicion of axillary lymph node involvement was confirmed with a core biopsy. Patients diagnosed with breast cancer (BC) and candidates for axillary lymph node dissection (ALND), based on the multidisciplinary oncologic committee, were considered for enrolment. All the patients had undergone a routine preoperative clinical and instrumental diagnostic assessment, including anamnestic data collection, blood exams, ECG, cardiologic and anesthesiologic evaluations, and thoracic X-rays.

After the referral for surgery, patients received a detailed explanation of the procedure and provided informed consent. All operations were performed by experienced surgeons with over 250 oncological breast procedures. Clinical data were collected in an electronic database and retrospectively analyzed.

### 2.3. Study Protocol

All patients received preoperatively a detailed US-ES, as mentioned below. During surgery, each patient undergoing ALND received an intraoperative detailed US-ES of each resected lymph node according to the 8 US-ES features analyzed. Subsequently, each lymph node was labeled and individually analyzed by a pathologist.

### 2.4. Preoperative and Intraoperative Ultrasound Protocol

#### 2.4.1. Preoperative US-ES

BC patients underwent US-ES scanning within 30 days before axillary surgery to assess the lymph node stage. High-quality US-ES images were acquired using Aixplorer^®^ Mach 30 (Supersonic Imagine) with a linear probe, L18-5 (centered at 10 MHz). Each patient was positioned supine with the arm raised above the head, and the entire axillary region was scanned. The examination was conducted by a sonographer with almost 10 years of experience in breast US.

#### 2.4.2. Intraoperative US-ES

ALND was carried out after the breast surgery, to remove all visible axillary lymph nodes (ALNs) in I and II Berg’s levels. US-ES scanning was performed immediately after the excision of lymph nodes and before the pathological evaluation. The dissected axillary specimens were cut into smaller pieces based on the palpation of nodes. If more than one node was observed within a piece of tissue, the piece was further subdivided with a scalpel so that only one node was visible per piece.

In the current study, 8 US-ES features were selected based on the BI-RADS lexicon and previous research.

1. Margins:
  - Definition: Well-demarcated and smooth margins are associated with normal lymph nodes, while an undefined margin (UM) predicts malignancy.
  - Scoring: Definite margins were assigned a value of 0, and UM was assigned a value of 1 [22].

2. Roundness Index (RI):
  - Definition: The RI is calculated as the longitudinal-to-transverse diameter ratio on the largest US section. It has been reported that RI > 1.5 is associated with benign diagnoses, while RI < 1.5 is associated with secondary lymphadenopathy [19].
  - Scoring: Thresholds for interpreting the RI will be empirically determined based on data obtained during the study. RI will then be classified as 1 or 0, depending on these cut-offs.

3. Cortical Thickness (CT):
  - Definition: The maximum CT on the largest US section was measured and recorded. A thickness >3 mm is considered for secondary malignancy, while <3 mm is considered for benign nodes [16].
  - Scoring: Thresholds for interpreting the RI will be empirically determined based on data obtained during the study. RI will then be classified as 1 or 0, depending on these cut-offs.

4. Echo Pattern:
  - Definition: Benign lymph nodes are slightly hypoechoic, while metastatic nodes appear markedly hypoechoic. This qualitative feature compares the US gray tone of the node to that of axillary fat.
  - Scoring: The marked hypoechogenic pattern (Hy) was classified as 1, and others as 0 [16].

5. Peripheral Vascularization:
  - Definition: Normal lymph nodes show hilar vascularization visible in Echo-color Doppler scans. The presence of cortical or capsular vessels is associated with malignancies.
  - Scoring: The presence of hilar vascularization was classified as 0, while the absence of vascularization or the visualization of more spots or peripheric vascularization (PV) received the value 1 [19].

6. SWE Hardness (SWEH):
  - Definition: ShearWave™ PL.U.S imaging measures non-invasive tissue stiffness in real time, expressed in kPa. Many studies identified the cut-off value of 150 kPa, suggesting that SWES > 150 kPa can be associated with secondary malignancy [23].
  - Scoring: Thresholds for interpreting the RI will be empirically determined based on data obtained during the study. RI will then be classified as 1 or 0, depending on these cut-offs.

7. Loss of fatty hilum (LFH):
  - Definition: Normal lymph nodes show as hilar hyperechogenic, correlated with the presence of the fatty hilum. The loss of contrast between the hypoechoic cortex and hyperechoic hilum, caused by hypertrophy of the former at the expense of the latter, is associated with a risk of metastasis [24].
  - Scoring: Marked LFH was classified as 1, and others as 0.

8. Peripheral infiltration (PI):
  - Definition: The presence of extracapsular infiltration or confluence tendencies is associated with an increased risk of metastasis [25].
  - Scoring: Marked PI was classified as 1, and others as 0.

These features were assessed during preoperative US evaluations using Aixplorer^®^ Mach 30 (Supersonic Imagine) with a linear probe, L18-5 (centered at 10 MHz). Each examination was conducted by a sonographer with nearly ten years of experience in breast US.

The 8 US-ES features were recorded. After the US-ES analysis, the specimens were placed into a saline solution in a plastic tray, labeled with an alphabetic character, and sent to the pathologist for histologic examination.

### 2.5. Statistical Analyses

The participants were divided into two groups, 50% were included in the training set and the remaining 50% in the validation cohort, to develop and test our algorithms. There were no significant differences in the characteristics of clinical and imaging data between the 2 groups. We reported the results for both cohorts and the whole sample. We employed an ROC curve analysis to measure the agreement between observed outcomes and predicted probabilities in both training and validation cohorts. This analysis also helped identify the optimal cut-off points for converting continuous variables into dichotomous variables and selecting the best predictors. The ROC curves allowed us to compute the AUC, which indicates the predictive performance of the algorithms, and create a clinical decision model based on a simple linear combination.

Our goal was to develop a simple clinical algorithm that can help clinicians in the decisional process of patients with BC and metastatic ALNs. To compare the quality of our algorithm, a machine learning technique was also applied, using two common classifiers: Naïve Bayes and a linear discriminant analysis (LDA). Naïve Bayes is a classification algorithm based on Bayes’ Theorem, with the assumption that predictors are independent. LDA is also a technique used for classification and dimensionality reduction. It aims to maximize the separability among known categories by projecting features into a lower-dimensional space. LDA is effective in settings where classes are linearly separable. We used malignancy as the outcome variable, continuous variables as covariates, and dichotomous variables as factors. For Naïve Bayes, the classifier selected the best predictors for inclusion based on the training set, which comprised half of the sample. For LDA, prior probabilities were estimated based on group sizes, and a pooled covariance matrix was utilized. We used leave-one-out cross-validation to assess the reliability of the results.

We used the risk factors that we identified to build a clinical algorithm. We split the sample into a training cohort (50%) and a validation cohort (50%) to develop and test our algorithms. We reported the results for both cohorts and the whole sample. We employed an ROC curve analysis to measure the agreement between observed outcomes and predicted probabilities in both training and validation cohorts. This analysis also helped identify the optimal cut-off points for converting continuous variables into dichotomous variables and selecting the best predictors. The ROC curves allowed us to compute the AUC, which indicates the predictive performance of the algorithms, and create a clinical decision model based on a simple linear combination.

We also carefully evaluated the performance of different models by calculating sensitivity, specificity, positive predictive value (PPV), negative predictive value (NPV), and overall correct classification. We developed a first model, Algorithm A, that maximizes the AUC. A second model, Algorithm B, combines a decision tree and aims to minimize the false negative rate. Therefore, we used a sensitivity threshold of at least 95% when converting continuous variables into dichotomous variables, instead of only optimizing the overall performance.

Moreover, we developed Algorithm C, an alternative model that does not rely on Ultrasound Elastography, taking into account varying resource availability. This approach ensured that surgeons who do not have this technology can still use our findings.

For each model, we ran an LDA classifier based on selected variables and a cut-off for that model to have a direct comparison for the clinical model.

**Figure 1:**
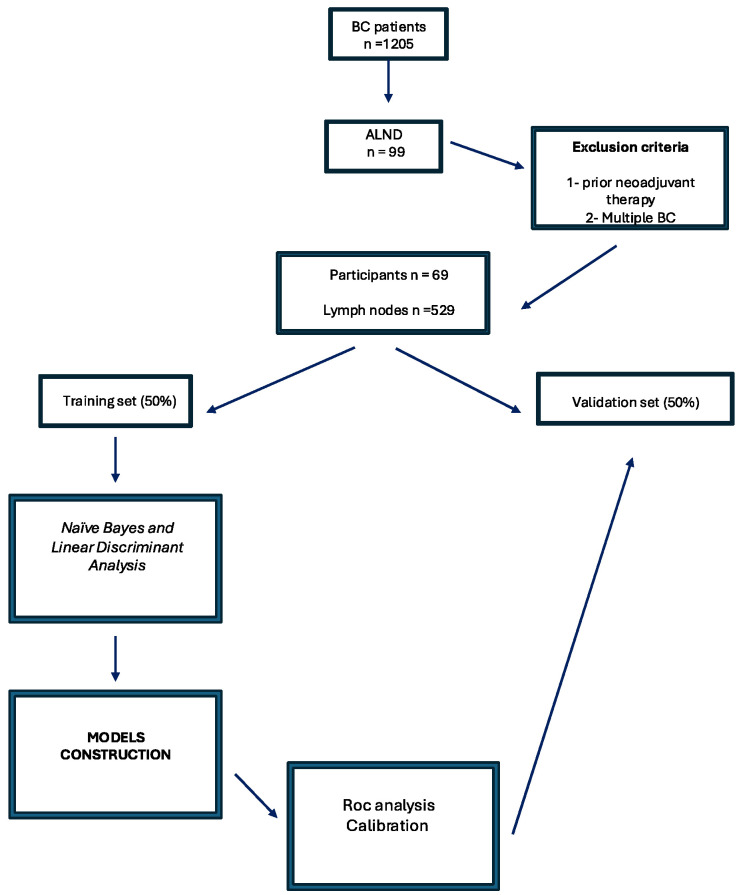
Workflow of study.

We coded categorical data as binary values (0 for absent, 1 for present). We used Bonferroni correction for multiple comparisons to test statistical significance and considered *p*-values < 0.006 as significant. Of the eight variables, five were dichotomous, and two of the three continuous variables did not satisfy the assumptions of parametric tests (as shown by Levene’s test). Therefore, we used the non-parametric Mann–Whitney U Test to compare the differences between the malignant and benign groups. We also computed non-parametric Spearman rank correlations among features to explore their relationships and guide model selection and development. We performed all data analyses using IBM (IBM Corp., Armonk, NY, USA, software version 29.0.1) (Figure 1).

### 2.6. Study Outcomes

The primary outcome was the construction of an innovative differentiation model of benign and malignant ALNs, which we will call the Cassandra score (CS), feasible for axillary US staging in patients with BC. The secondary outcome was the creation of a further predictive model also widely available in US machines without ES.

## 3. Results

### 3.1. Study Population

From 1 January 2017 to 1 January 2023, 2429 patients were referred to the Surgery and Oncological Unit of Campania University “Vanvitelli” (Naples, Italy) and the Breast Surgery Unit of “Cobellis” Hospital (Salerno, Italy) for breast disease. Of these, 1205 were diagnosed with BC. Each case was reviewed by a multidisciplinary oncological group. Ninety-nine women with BC were initially considered for ALND, and 69 met the inclusion criteria and were included in the study. The average age of the participants was 55.3 ± 5.3 years, with a mean BMI of 26.8 ± 3.7 kg/m^2^.

A total of 529 lymph nodes were resected and examined using US-ES. Among these, 236 (44.6%) exhibited indefinite margins, 278 (55.5%) displayed a marked hypoechoic pattern, 183 (35.6%) showed hilar vascularization, 207 (39.1%) had loss of the fatty hilum, and only 23 (4.3%) nodes infiltrated the peripheral tissue. The average cortical thickness was 3.6 ± 0.3 mm, and the shear wave (SW) value was 141.12 ± 21.7. The average roundness index was 1.7 ± 0.5. Out of these lymph nodes, 209 (39.5%) were confirmed to be metastatic.

### 3.2. Study Outcomes

Our comprehensive analysis identified statistically significant differences between benign and malignant lymph nodes (*p* ≤ 0.002) across all examined features. However, there was an overlap in these features, except for peripheral infiltration, which was exclusively present in malignant samples (as indicated in Table 1). Notably, significant differences were observed in features including the SWE Hardness (SWEH), roundness index (RI), loss of the fatty hilum (LFH), indefinite margins (IM), Hypoechogenicity (Hy), cortical thickness (CT), peripheral infiltration (PI), and Peripheral Vascularization (PV).

A further analysis using Spearman correlations among features and their associations with malignity status confirmed the ranking observed in the Mann–Whitney U Test, with all correlations being statistically significant (*p* < 0.01). The correlation coefficients (*r_s_*) ranged from 0.789 for SWE Hardness to 0.132 for PV. We observed medium-to-low correlations among various features, with *r_s_* ranging from 0.583 (between SWEH and Negative RI) to zero. The sole negative correlation (*r_s_* = −0.170) was identified between Hy and PV. Additionally, partial correlations controlled for malignancy (equivalent to the pooled within-groups matrix of the linear discriminant analysis) revealed minimal-to-negligible associations among variables, ranging from 0.258 (between SWEH and RI) to zero. Notably, the only significant negative correlation (*r_s_* = −0.270) persisted between Hy and PV (as presented in Table 2).

#### 3.2.1. Classifiers

In the evaluation of the Naïve Bayes classifier, lymph nodes were evenly split into a training and a validation group, each comprising half of the total. The classifier achieved an overall prediction accuracy of 94.4% for the training group and 94.6% for the validation group. When applied to the entire dataset, the classifier maintained a high accuracy rate of 94.9%.

For the LDA conducted on the original group, the overall accuracy was 93.4%. Cross-validation slightly reduced the accuracy to 93.0%. Detailed metrics such as sensitivity, specificity, positive predictive value (PPV), and negative predictive value (NPV) are presented in Table 3. The analysis revealed a canonical correlation of 0.841 for the canonical function, with standardized canonical discriminant function coefficients ranging from 0.604 for SWEH to −0.029 for PV. These standardized coefficients are detailed in Table 4.

#### 3.2.2. ROC Analysis

All models utilized the same training and validation sets. Initially, an ROC analysis was conducted on the training set for each feature individually to select features for the final model and establish variable cut-offs. The AUC was above 0.747 and statistically significant (*p* < 0.001) for all variables except for PI, with an AUC of 0.558 (*p* = 0.118), and PV, with an AUC of 0.581 (*p* = 0.025). Both the LDA model and ROC analysis, along with z-scores from the Mann–Whitney U Test, yielded a consistent variable ranking. However, the Model Quality (MQ) was considered low for peripheral infiltration (MQ = 0.49) and PV (MQ = 0.51). For Model A, cut-offs that optimize Youden’s Index were applied, resulting in a cut-off of 142 for SWEH, −1.55 for Negative RI, and 3.50 for CT.

#### 3.2.3. Calibration and Validation of Clinical Algorithm

##### Model A

Following the conversion of continuous variables to binary ones based on the ROC analysis cut-offs, and after removing the two variables that were not statistically significant, we developed a straightforward algorithm that employs a linear combination of the newly binary variables. These variables are denoted with a “*B*” suffix to indicate their binary nature. To facilitate easy recall and application, we opted to use only integers as coefficients in the combination. Considering the robust predictive ability of SWEH and RI even when used independently, we assigned them a weight of 2. The other variables were assigned a weight of 1 due to their comparably strong predictive power. Consequently, the formula for calculating the Lymph Node Malignity Score (LNMS) is as follows:

LNMS_A_ = 2 × BSWEH_A_ + 2 × BRI_A_ + LFH + UM + Hy + BCT

The LNMS_A_ can range from 0 to 8, with a score of 8 indicating a higher likelihood of malignancy.

The ROC analysis for Model A on the training set revealed an impressive AUC of 0.978 (S.E. = 0.007, *p* < 0.001), a Gini Index of 0.956, and a maximum Kolmogorov–Smirnov (max K-S) statistic of 0.853 at a cut-off of 3.50. The model’s overall quality was rated at 0.96. It achieved an overall prediction accuracy of 92.1% in the training set, 88.3% in the validation set, and 90.2% across the entire sample. The NPV in the validation set stood at 95.1%.

##### Model B

In Model B, we altered the cut-offs of SWEH and RI to reduce the risk of false negatives (i.e., maximizing NPV), setting sensibility to at least 0.95, leading to a cut-off of 137 for SWEH, −2.25 for Negative RI, and 3.50 for CT.

Consequently, the formula for calculating the LNMS is as follows:

LNMS_B_ = 2 × BSWEH_B_ + 2 × BRI_B_ + LFH + UM + Hy + BCT

The ROC analysis for Model B on the training set revealed an impressive AUC of 0.973 (S.E. = 0.009, *p* < 0.001), a Gini Index of 0.863, and a max K-S statistic of 0.863 at a cut-off of 4.50. The model’s overall quality was rated at 0.95. It achieved an overall prediction accuracy of 92.1% in the training set, 88.6% in the validation set, and 90.4% across the entire sample. The NPV in the validation set stood at 99.2%.

##### Model C

Model C uses the same cut-offs as Model B, but BSWEH_B_ is not included in the formula.

Consequently, the formula for calculating the LNMS is as follows:

LNMS_C_ = 2 × BRI_B_ + LFH + UM + Hy + BCT

The LNMS_C_ can range from 0 to 6, with a score of 6 indicating a higher likelihood of malignancy.

**Figure 2:**
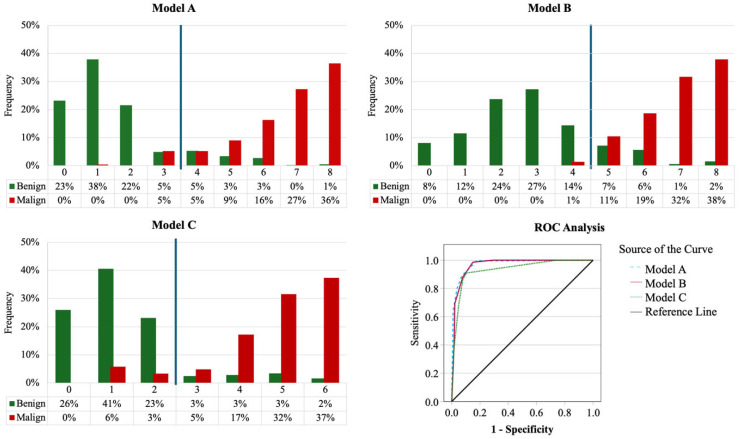
Percentages of benign and malign cases for each value of the model function LNMS and ROC analysis comparison. The vertical blue line is the cut-off for each model. Green represents benign and red represents malignant lymph node frequency for each value of LNMS. In the bottom-left quadrant is the ROC for each model.

The ROC analysis for Model C on the training set revealed an impressive AUC of 0.940 (S.E. = 0.015, *p* < 0.001), a Gini Index of 0.879, and a max K-S statistic of 0.817 at a cut-off of 2.50. The model’s overall quality was rated at 0.91. It achieved an overall prediction accuracy of 90.9% in the training set, 89.4% in the validation set, and 90.2% across the entire sample. The NPV in the validation set stood at 89.4%. Figure 2 presents a comparison of the models.

We performed LDA with the variables included in Models A, B, and C, to compare the performance of simplified models with a linear combination of integer coefficients for each feature, with models optimized to have the optimal coefficients for the discriminant function. Each LDA model is reported in Table 3 below the corresponding clinical model. There is no significant difference between the clinical and corresponding LDA models in the overall accuracy if we compare the whole sample. LNMS_A_ accuracy = 0.902, LDA_A_ accuracy = 0.919; LNMS_B_ accuracy = 0.904, LDA_B_ accuracy = 0.907; LNMS_C_ accuracy = 0.902, LDA_C_ accuracy = 0.877.

#### 3.2.4. Two-Tier Model: Cassandra Score

**Figure 3:**
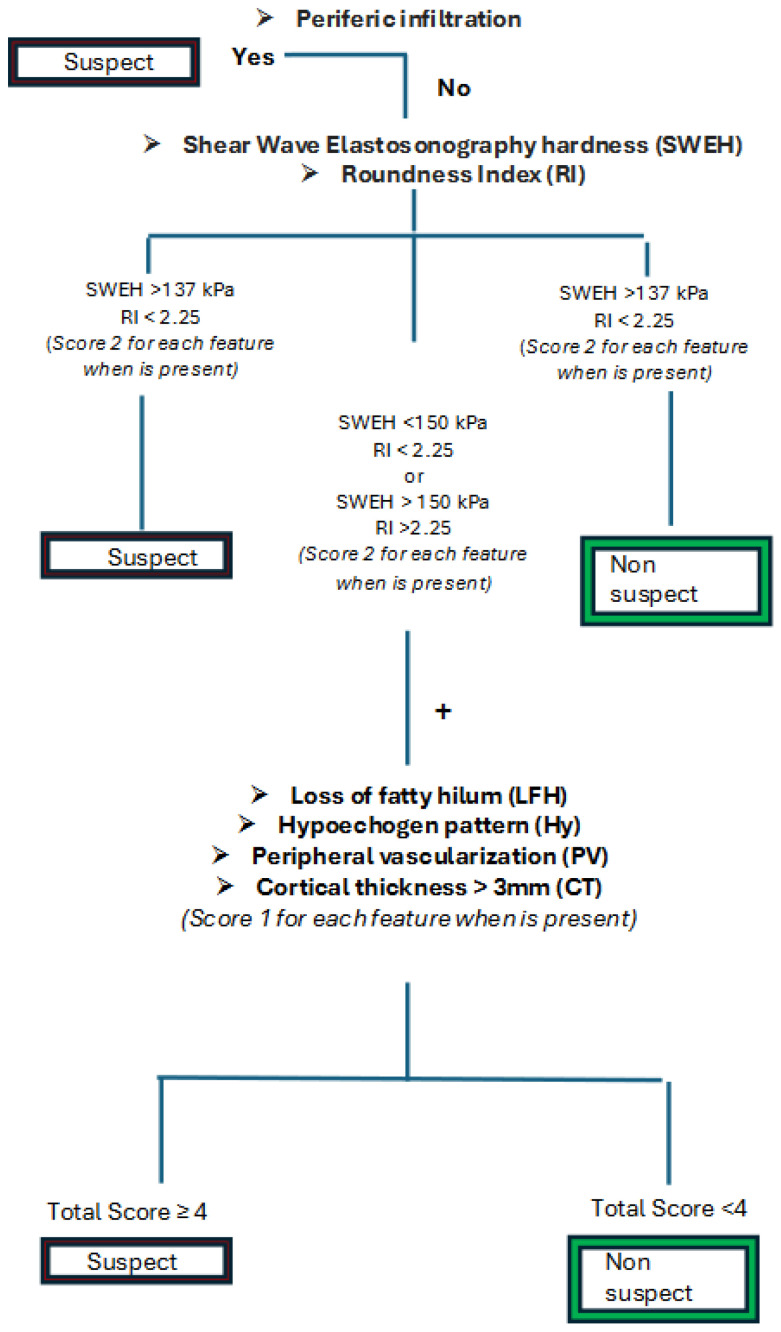
Cassandra score algorithm.

We proposed a two-tiered model leveraging the strong predictive capabilities of BSWEH_B_ and BRI_B_. In the first tier, only BSWEH_B_ and BRI_B_ were evaluated: a positive result was predicted if both were positive and conversely, a negative result was predicted if both were negative. In the second tier, if there was a mix of positive and negative results between BSWEH_B_ and BRI_B_, we proceeded with the algorithm in Model B (Figure 3).

The model demonstrated an overall prediction accuracy of 90.2% in the training set, 87.5% in the validation set, and 88.9% across the entire dataset. The NPV was notably high at 99.2% in the validation set. Notably, 44% of the total sample was classified in the first tier.

It is important to highlight that while PI exhibited limited overall predictive power, it had a significant PPV. PI was found in only 23 lymph nodes, representing 4.35% of the total sample, yet all lymph nodes with PI were malignant, yielding a PPV of 100%. However, the malignancy of all ALNs with PI was already correctly predicted by Models A and B, and the two tiers were already computed in the first tier. Model C missed only one lymph node with PI (Table 4).

## 4. Discussion

The detection of ALN metastasis is crucial in managing patients with primary BC. Currently, SLNB is the most accurate method for axillary staging, especially in cases of BC with negative axillary US, as strongly suggested by Veronesi et al. SLNB is preferred over ALND due to its elevated accuracy (93–97%), low false negative rate (i.e., 9.8%), and consistent reduction in morbidities [26]. Recent clinical trials, such as ACOSOG Z0011, have explored the potential for de-escalating axillary surgery, finding no additional benefit of ALND in patients with one or two metastases detected by SLNB [27]. Imaging plays a critical role in selecting patients with suspected axillary involvement who could be candidates for neoadjuvant therapy or ALND [28]. US is the tool of choice for ALN assessment [29,30], despite it being operator-dependent. BI-RADS was introduced to guide the risk classification of breast lesions, but the interpretation of each feature remained operator-dependent and subjective, leading to inter- and intra-observer variability in reproducibility. Many authors have proposed multiple strategies to standardize the evaluation of US characteristics, developing nomograms based on key features [31,32]. For example, Yan et al. set up a nomogram model based on five features of BC lesions. The method was compared to the BI-RADS model, demonstrating satisfactory discriminative function (area under the receiver operating characteristic [ROC] curves [AUC], 0.940; 95% confidence interval [CI], 0.909 to 0.971; sensitivity, 0.905; and specificity, 0.902 in the training cohort and AUC, 0.968; 95% CI, 0.941 to 0.995; sensitivity, 0.971; and specificity, 0.867 in the validation cohort) [33]. Another interesting approach consisted of the construction of a prediction model for the ALN status of BC patients based on a radiomics analysis of US images of primary breast lesions. The trial demonstrated high accuracy in the malignancy prediction (AUC of 0.846 (95% CI, 0.790–0.902) for the training cohort and 0.733 (95% CI, 0.613–0.852) for the validation cohort) [34]. Other authors who developed similar models capable of predicting ALN status by examining the US features of BC lumps reported analog results [35,36,37,38].

To the best of our knowledge, the current study is the first to analyze ALN features with US-ES to identify a reliable system capable of standardizing the axillary staging in patients with BC. The main reported limitation in this type of assessment was to correlate the ALN identified in axillary scans with a definitive histological examination. Therefore, we decided to perform intraoperative US-ES (immediately after ALND), to classify and send each lymph node individually for definitive histological examination. Therefore, we established a direct correlation between the US-ES characteristics and the malignancy or benignity of the ALN. A total of 529 lymph nodes were resected and examined using US-ES. We developed several statistical models to identify the most clinically functional approach for this kind of patient. In Model A, we achieved a positive predictive value (PPV) of 95.1%, while in Model B, we focused on diagnostic sensitivity, reaching a PPV of 99.1%. Indeed, we deemed it clinically desirable to minimize false negatives as much as possible. Model C was developed by excluding the extensive use of ES, considering that this technique is not available on all US machines. In both models, A and B, the roundness index (RI) and SWEH were found to have a high impact, so much so that they were assigned a weight of 2, while the other variables were assigned a weight of 1. In addition, we developed a final model, which probably has the best performance in clinical practice. Considering the important impact of SWEH and RI, if both these variables are positive, the ALN is considered strongly suspicious, and a further analysis of the remaining US features is unnecessary. However, if only one of the variables is positive, the calculation of the score proceeds. This latter procedure represents the final Cassandra score.

A further interesting focus is the potential changes in lymph node morphology after lymphoscintigraphy in patient candidates for SLNB, following tracer accumulation. The argument is complex, and no previous studies reported a clear indication of the topic. However, we avoided enrolling SLNB patients in the study, excluding the possible effects of the bias.

The main limitations of the current study are the retrospective nature and the sample size. Another possible problem is the duration of the US-ES examination after the excision because the delayed immersion in fixative liquids can be associated with specimen artifacts [39]. Furthermore, we have not considered the use of CEUS, which appears promising and interesting as reported in a recent meta-analysis [40]. Moreover, we verified that the examinations were concluded in less than 40 min, avoiding reaching the limits of an hour for the delay.

## 5. Conclusions

The prediction of malignancy of ALN is a cornerstone in the staging of patients with BC. Several methods have been proposed in the literature without reaching satisfactory results. The Cassandra score is a simple, non-invasive, fast, and reliable method that showed a PPV of 99.1% in the malignancy prediction of ALN, potentially being also well suited for young sonographers. Future studies with a larger sample size are desirable, also being in a prospective setting. A further study, focusing on validating the score in a preoperative setting, is ongoing.

## Figures and Tables

**Table 1:** Descriptive Statistics of Initially Selected Variables.

Variable	Malignity	Cases	Mean	SD	Median	Range	Mann-Whitney U Test
SWE Hardness	0	320	131.00	11.89	130.00	110–160	-
	1	209	161.73	14.08	160.00	110–198	*z* = 17.21, *p* < 0.001
Negative Roundness Index	0	320	−2.02	0.43	−1.90	−2.9–−1.1	-
	1	209	−1.35	0.34	−1.30	−2.8–−1.0	*z* = 15.81, *p* < 0.001
Loss of Fatty Hilum	0	320	0.15	0.36	0	0–1	-
	1	209	0.76	0.43	1	0–1	*z* = 13.88, *p* < 0.001
Indefinite Margins	0	320	0.25	0.43	0	0–1	-
	1	209	0.75	0.43	1	0–1	*z* = 11.40, *p* < 0.001
Hypoechogenicity	0	320	0.33	0.47	0	0–1	-
	1	209	0.82	0.38	1	0–1	*z* = 11.13, *p* < 0.001
Cortical Thickness	0	320	3.20	1.37	3.00	1–7	-
	1	209	4.41	1.17	5.00	1–7	*z* = 9.76, *p* < 0.001
Peripheral Infiltration	0	320	0	0	0	0–1	-
	1	209	0.11	0.31	0	0–1	*z* = 6.06, *p* < 0.001
Peripheral Vascularization	0	320	0.30	0.46	0	0–1	-
	1	209	0.43	0.50	0	0–1	*z* = 3.04, *p* = 0.002

**Table 2:** Pooled Within-Groups Correlation Matrix.

Features	SWE Hardness	Negative Roundness Index	Loss of Fatty Hilum	Indefinite Margins	Hypoechogenicity	Cortical Thickness	Peripheric Infiltration	Peripherical Vascularization
SWE Hardness	1.000	0.108 *	0.258 **	0.042	0.108 *	0.179 **	0.071	0.191 **
Negative Roundness Index	0.108 *	1.000	0.174 **	0.061	0.123 *	0.101 *	0.004	0.032
Loss of Fatty Hilum	0.258 **	0.174 **	1.000	−0.031	0.166 **	0.135 **	0.015	−0.081
Indefinite Margins	0.042	0.061	−0.031	1.000	−0.011	0.106 *	0.061	0.108 *
Hypoechogenicity	0.108 *	0.123 **	0.166 **	−0.011	1.000	−0.037	0.024	−0.270 **
Cortical Thickness	0.179 **	0.101 *	0.135 **	0.106*	−0.037	1.000	−0.018	0.130 **
Peripheral Infiltration	0.071	0.004	0.015	0.061	0.024	−0.018	1.000	0.126 **
Peripheral Vascularization	0.191 **	−32	−0.081	0.108*	−0.270 **	0.130 **	0.126 **	1.000

** Correlation is significant at the 0.01 level (2-tailed). * Correlation is significant at the 0.05 level (2-tailed).

**Table 3:** Model Accuracy.

Model	Set	Sensitivity	Specificity	PPV	NPV	Accuracy
**Naive Bayes**	Training	0.911	0.965	0.939	0.948	0.944
	Validation	0.898	0.980	0.970	0.931	0.946
	Whole Sample	0.923	0.966	0.946	0.951	0.949
**LDA**	Original	0.914	0.947	0.918	0.944	0.934
	Cross-Validated	0.914	0.941	0.910	0.944	0.930
**Model A**	Training	0.952	0.901	0.891	0.967	0.921
	Validation	0.933	0.849	0.803	0.951	0.883
	Whole Sample	0.943	0.875	0.831	0.959	0.902
LDA: model A	Original	0.890	0.934	0.899	0.929	0.919
	Cross-Validated	0.885	0.928	0.889	0.925	0.911
**Model B**	Training	0.981	0.883	0.843	0.986	0.921
	Validation	0.991	0.818	0.782	0.992	0.886
	Whole Sample	0.986	0.850	0.811	0.989	0.904
LDA model B	Original	0.914	0.903	0.860	0.941	0.907
	Cross-Validated	0.904	0.863	0.811	0.932	0.879
**Model C**	Training	0.904	0.913	0.870	0.936	0.909
	Validation	0.914	0.881	0.940	0.894	0.894
	Whole Sample	0.909	0.897	0.852	0.938	0.902
LDA model C	Original	0.833	0.906	0.853	0.892	0.877
	Cross-Validated	0.833	0.906	0.853	0.892	0.877
**Two-tier**	Training	0.981	0.851	0.810	0.986	0.902
	Validation	0.991	0.799	0.765	0.992	0.875
	Whole Sample	0.986	0.825	0.786	0.989	0.889

**Table 4:** Feature Weights for Different Models.

Features	LDA	Training Set ROC Analysis					
	Standardized Canonical Discriminant Function Coefficients	AUC	S.E.	Gini Index	Max K-S	Cut-off	Model Quality **
SWE Hardness	0.604	0.939 *	0.018	0.879	0.808	142.00	0.90
Negative Roundness Index	0.373	0.892 *	0.023	0.784	0.705	−1.55	0.85
Loss of Fatty Hilum	0.226	0.796 *	0.030	0.591	0.591	0.50	0.74
Indefinite Margins	0.315	0.748 *	0.032	0.495	0.495	0.50	0.69
Hypoechogenicity	0.204	0.760 *	0.030	0.520	0.520	0.50	0.70
Cortical Thickness	0.097	0.759 *	0.030	0.518	0.465	3.50	0.70
Peripheral Infiltration	0.110	0.558	0.037	0.115	0.115	0.50	0.49
Peripheral Vascularization	−0.029	0.581	0.036	0.162	0.162	0.50	0.51

* Correlation is significant at the 0.001 level (2-tailed). ** A good model has a value above 0.5. A value less than 0.5 indicates the model is no better than random prediction.

## Data Availability

The raw data supporting the conclusions of this article will be made available by the authors, without undue reservation.
